# Women's attitude towards wife-beating and its relationship with reproductive healthcare seeking behavior: A countrywide population survey in Bangladesh

**DOI:** 10.1371/journal.pone.0198833

**Published:** 2018-06-07

**Authors:** Md. Nuruzzaman Khan, M. Mofizul Islam

**Affiliations:** 1 Department of Population Sciences, Jatiya Kabi Kazi Nazrul Islam University, Mymensingh, Bangladesh; 2 Department of Public Health, La Trobe University, Melbourne, Australia; Tokyo Medical and Dental University, JAPAN

## Abstract

**Background:**

Intimate partner violence (IPV) is a global public health problem that has substantial consequences on the physical, mental, sexual and reproductive health of women. This study examined the association between women’s attitudes towards wife-beating and their utilization of reproductive healthcare services.

**Method:**

Two waves of Bangladesh Demographic and Health Survey data were analyzed using multivariate regression. Outcome variables were a set of reproductive healthcare services, namely contraception use, modern contraception use, antenatal visit by skilled health professionals (SHP), delivery in healthcare facilities, delivery by SHP and postnatal check up by SHP. Attitudes towards abuse were assessed by a set of five questions that asked the situation under which ‘hitting or beating’ one’s wife is justifiable.

**Results:**

Around 32% of the participants reported that hitting or beating wife by husband was justified in certain situations. There is a gradient in the relationship between number of healthcare services accessed and number of situations justified for beating wife. Women who strongly reject the justification of wife beating were more likely than those who reject that weakly to report contraception use, antenatal care by SHP, delivery in healthcare facilities, delivery care by SHP, and postnatal care by SHP.

**Conclusions:**

Women’s attitudes towards ‘wife beating’ have a significant association with reproductive healthcare seeking behavior. The impact of this malpractice on women’s health and consequences thereafter need to be brought in the forefront of public health campaign.

## Introduction

Intimate partner violence (IPV) is a well-recognized public health problem [1, 2]. Women who are victims of IPV have increased risk of unintended pregnancy, multiple abortions and reduced sexual autonomy [3]. Additionally, IPV during pregnancy may significantly increase the risk of preterm delivery, low birth weight infant and neonatal death [3–5]. IPV is also associated with mental health problems such as depression, suicide, posttraumatic stress disorder (PTSD) [6, 7] and other adverse health outcomes including chronic fear and cardiac problems [8]. Primary healthcare has always been considered important, aiming to provide a safe environment where abused women can confidentially disclose experiences of violence and receive care for adverse health outcomes caused by violence [9]. This care is important particularly for pregnant women. However, although this sounds reasonable, women who are victims may not consider accessing healthcare due to perceived ‘normality’ of IPV, its confidential nature, and/or fear of consequences [10]. In a developing country such as Bangladesh, women’s reluctance toward accessing healthcare may have substantial public health implications, mainly due to the fact that overall utilization of reproductive healthcare still remains low. For instance, a little more than half (54%) of the women used modern contraception, 64% used antenatal care, and only 37% delivered in healthcare facilities [11].

In many developing countries including Bangladesh, there is a general acceptance of ‘wife-beating’–a common type of IPV–often perpetuated by the commonly held norms and gender roles in society [12]. For instance, it is generally believed that a man has the right to assert power over a woman and correct female behavior [13, 14] using physically punitive measures such as beating [15]. A woman’s attitude toward wife-beating is considered a proxy for her perception of her status [11, 16]. A woman who considers such violence ‘unjustifiable’ is likely to be aware of her greater sense of entitlement, self-esteem, status, and to reflect positively on her sense of empowerment [17–19]. On the other hand, a woman who considers such violence ‘justifiable’ accepts the right of her husband to control her behavior even by means of violence [11, 18, 19]. A direct relationship exists between the tolerant attitudes toward violence against women and the actual occurrence of violence against women [20–22]. A tolerant attitude toward violence may arise from the fact that the violence is considered a normal phenomenon of a woman’s life [23], and/or the woman may have a lower sense of entitlement or self-esteem [24]. This normality and/or lower sense of entitlement or self-esteem may act as a barrier to accessing medical care, even when ideally care is required–particularly during the reproductive period. Therefore, we hypothesize that women with a tolerant attitude towards violence may use reproductive healthcare services less than those who do not hold such an attitude. Using nationally representative Bangladesh Demographic and Health Survey (BDHS) data we examined the relationship between women’s attitudes towards *wife-beating* and their healthcare seeking behavior.

## Method

### Data

We used aggregate data of two waves of BDHS, collected in 2011 and 2014. The survey was based on a two-stage stratified sample of households whereby enumeration areas (clusters) were first drawn from the national population and housing census sampling frame conducted in 2011 by Bangladesh Bureau of Statistics. In the first stage of sampling, 600 primary sampling units were selected with probability of selection proportional to the unit size. In the second stage, 30 households were selected within each primary sampling unit by systematic random sampling. Further details of sampling design and data collection approach of BDHS can be found elsewhere [11, 16]. In two waves, a total of 35,705 ever-married women were interviewed. The response rates in both waves were more than 98%. The questions on domestic violence were administered only on one ever married woman (age 15–49 years) per household. Selecting only one person to respond to IPV-related questions protected the privacy of the person and helped ensure the other respondents in the household were not aware of the types of questions that the selected respondent was asked. If privacy could not be ensured, the interviewers were instructed to skip the module. If there was more than one eligible women in the household, the respondent was selected randomly through a specially designed simple selection procedure based on the Kish Grid [25]. Using this method, a total of 16,639 (46.60%) women were interviewed for IPV during two waves of the survey. Among these women, a total of 9,632 reported at least one birth within three years preceding the survey and were considered eligible for this study. The survey protocol was reviewed and approved by the National Research Ethics Committee in Bangladesh. Because the existence of a signed consent form can provide a risk in itself for the abused person, only oral informed consent was obtained from the respondents. The ethics committee approved this consent procedure. All data were fully anonymized by the BDHS authority prior to making them available.

### Exposure variable

We developed composite scores of women’s attitudes towards justification of wife-beating based on women’s response to a set of five questions regarding the conditions under which hitting or beating one’s wife would be justifiable. Five questions are: 1) if she goes out without telling her husband; 2) if she argues with her husband; 3) if she neglects the children; 4) if she refuses to have sex with her husband; and 5) if she burns food. For each of these questions, response options to whether hitting or beating (used as beating hereafter) would be justified in this situation were yes (score = 1) or no (score = 0). Composite scores were computed for each respondent based on the average of responses to the five items mentioned above. We then split the mean score into three categories: i) reject weakly (score 1.00–0.68), ii) reject moderately (score 0.67–0.34), and iii) reject strongly (score 0.33–0). Therefore, a high mean score indicates a weak rejection (i.e. a strong justification for beating) and a low mean score indicates a strong rejection (i.e. a weak justification for beating).

### Outcome variables

The utilization of a range of reproductive healthcare services were the outcome variables. They were, namely (i) the types of contraception methods respondents used (traditional *vs* modern); (ii) number of times they received antenatal care; (iii) types of services respondents accessed for antenatal and postnatal care; (iv) place of delivery (home or healthcare facility); and (v) whether the respondents received healthcare from skilled health professionals (SHPs) during their recent delivery. We categorized responses to these questions as either ‘yes’ or ‘no’. Traditional contraception includes periodic abstinence, withdrawal and folk methods while modern contraception includes pill, female- and male-sterilization, intrauterine devices, injectable, implants, male and female condom, diaphragm, and emergency contraception. SHPs refer to doctor, nurse/midwife/paramedic, skilled birth attendant, family welfare visitor, community skilled birth attendant, medical assistant or sub-assistant community medical officer.

### Statistical analysis

Descriptive statistics were used to estimate the demographic characteristics of participants and their attitudes towards wife-beating in particular circumstances. To examine the relationship of these attitudes with healthcare seeking and or usage behavior, we estimated both unadjusted and adjusted associations using multivariate logistic regression. A range of socio-demographic covariates that were found important in the literature and could be consistently measured in two waves of survey were included in multivariate models. The variables were, *namely* the maternal age at birth, place of residence (urban, rural), region of residence (seven divisions: Barisal, Dhaka, Chittagong, Khulna, Rajshahi, Rangpur, Sylhet), wealth quintile (poorest, poorer middle, richer, richest), years of education for women and their husbands, and the survey years. In BDHS, individuals were nested in households, and households were nested in communities. Thus, individuals in the same household and households in the same community were strongly clustered. We used Stata’s ‘*svy*’ command in all analyses for controlling the effect of this complex survey design. Statistical software Stata version 15 (StataCorp. LP, College station, USA) was used for analysis.

## Results

**Fig 1:**
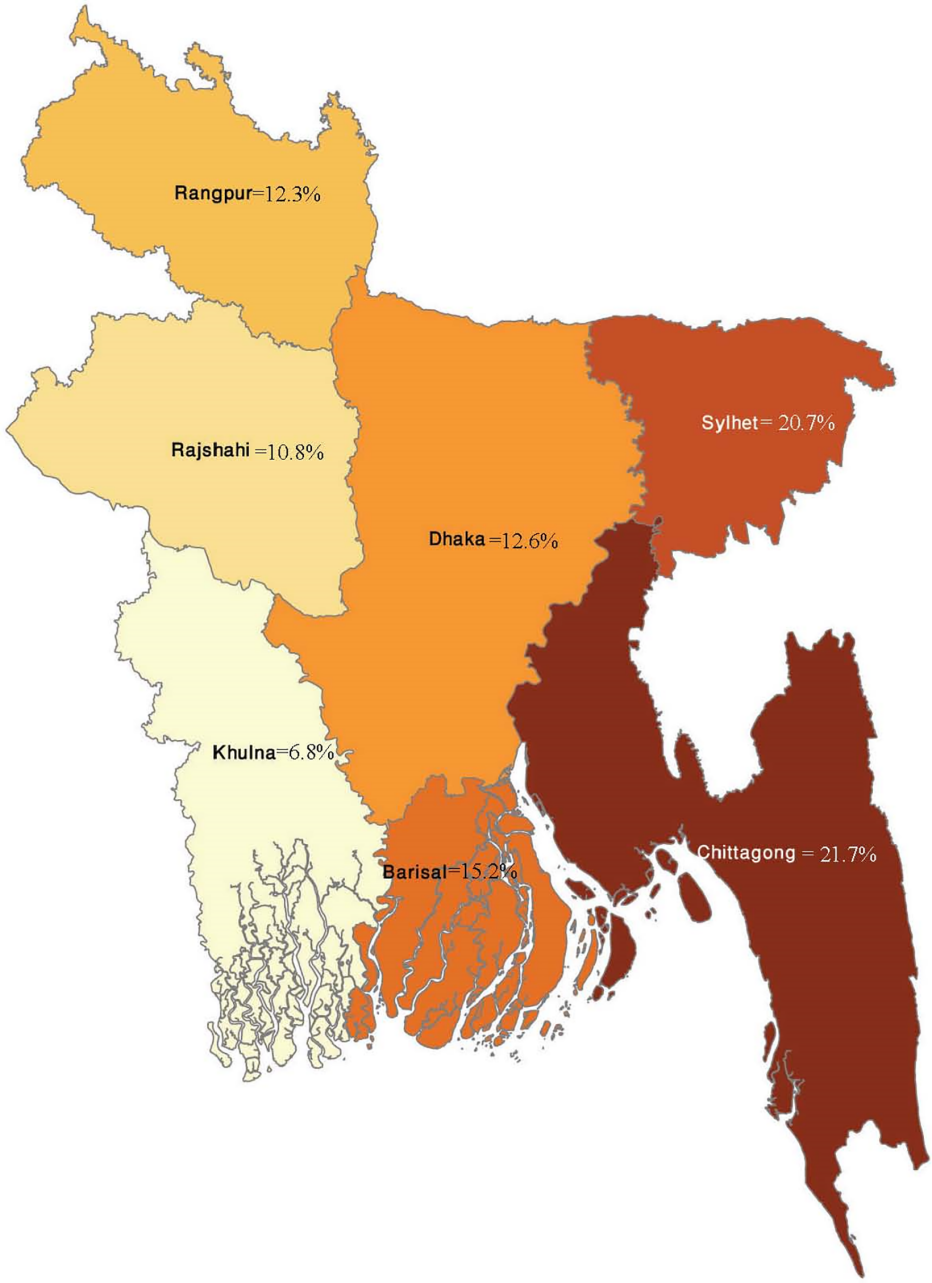
Spatial variation in percentages of women who weakly reject wife-beating by husband.

**Fig 2:**
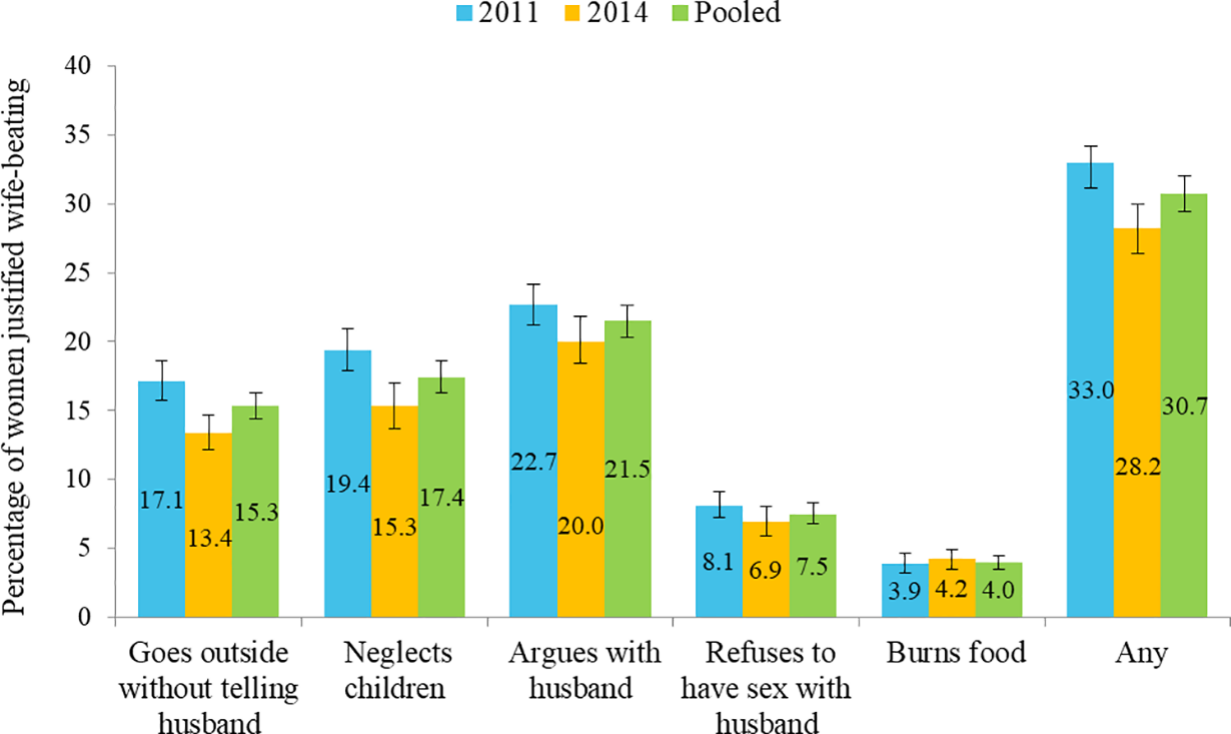
Respondents’ perceptions of the situations justified for beating wife.

A total of 9,632 women who responded the questions on perception regarding wife-beating and gave at least one live birth within three years preceding the survey were included in this study. The shares of 2011 and 2014 survey wave were 51.3% (4,944) and 48.7% (4,688) respectively. There were no significant differences in socio-demographic characteristics between women who did and did not respond to the questions about wife-beating. The mean age of respondents was 26 years, mean years of schooling was approximately six years. Overall, 32% of respondents justified wife-beating for at least one of the given reasons, and 2% justified it for all five reasons. However, the proportions of women who weakly rejected several causes of beating by husband were not consistent across regions (Fig 1). Almost 22% of the respondents thought beating is justified if they argue with their husband, followed by neglecting children (17.4%) and going out without telling husband (15.3%). The percentages of women who believed beating is justified are presented in Table 1 and Fig 2.

**Table 1:** Descriptive statistics on the demographic characteristics of participants and their overall attitude towards wife-beating.

*Variable*	Statistic
***Demographic characteristics of mothers (n = 9632)***	
Mean age (95% CI)	25.6 (25.04–25.08)
Mean year of education (95% CI)	5.8 (5.04–6.03)
Child birth order (95% CI)	2.5 (2.46–2.54)
***Exposure variables*: *perceptions regarding wife-beating***	
Beating justified if wife goes outside without telling husband, % (95% CI)	15.3 (14.4–16.3)
Beating justified if wife neglects children, % (95% CI)	17.4 (16.3–18.6)
Beating justified if wife argues with husband, % (95% CI)	21.5 (20.3–22.7)
Beating justified if wife refuses to have sex with husband, % (95% CI)	7.5 (6.8–8.3)
Beating justified if wife burns food, % (95% CI)	4.0 (3.5–4.5)
Beating justified for any of the above five reasons, % (95% CI)	
Reject strongly, % (95% CI)	81.6 (80.4–82.7)
Reject moderately, % (95% CI)	13.8 (12.9–14.7)
Reject weakly, % (95% CI)	4.6 (4.0–5.2)
***Outcome variables***	
Any contraception use, % (95% CI)	92.8 (92.0–93.6)
Modern contraception use, % (95% CI)	65.1 (63.7–66.5)
ANC ≥1 visit by SHP, % (95% CI)	47.1 (45.5–48.7)
ANC ≥4 visit by SHP, % (95% CI)	24.2 (22.8–25.6)
Place of delivery, % (95% CI)	29.5 (28.0–31.0)
Delivery by SHP, % (95% CI)	26.7 (25.4–28.0)
Mothers' postnatal checkup by SHP, % (95% CI)	24.6 (23.3–25.9)

Note. SE: standard error, CI: confidence interval, ANC: antenatal care, SHP: skilled health professionals

Contraception use was reported by 93% of the participants, of which 65% reported use of modern contraception. Around one-fourth of the respondents reported they received more than four antenatal visits (24.2%), postnatal checkup by SHP (24.6%), and delivery by SHP (26.7%). Almost half (47.1%) of the respondents received ANC from SHP at least once, and a quarter reported receiving postnatal checkup by SHP. There remains regional variation in utilization of reproductive healthcare for each of these services.

**Fig 3:**
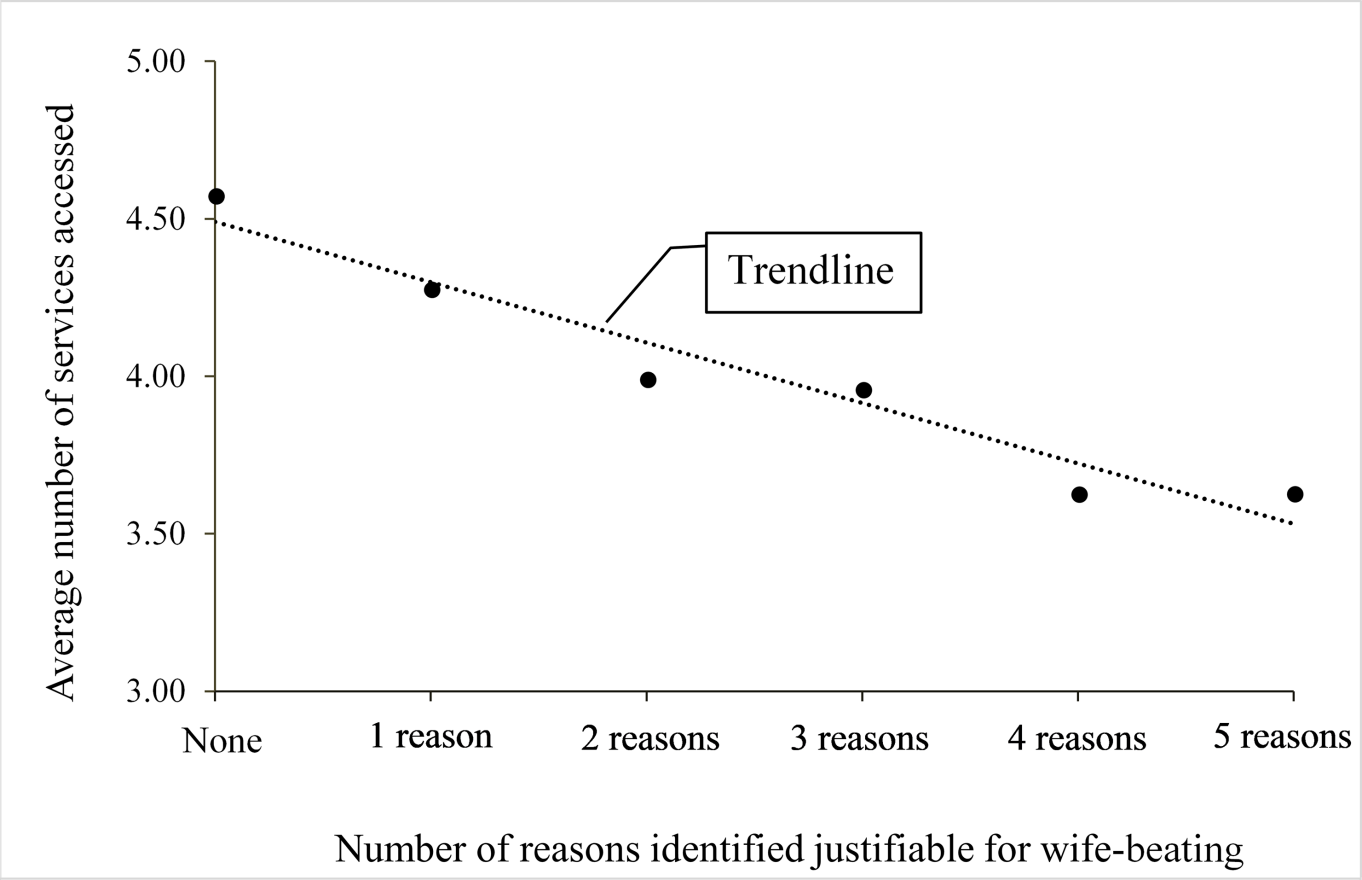
Trend in utilisation of reproductive healthcare services across number of reasons identified justifiable for wife-beating.

There is a gradient in the relationship between number of services accessed and number of reasons justified for wife-beating (Fig 3). A higher number of women who reported no justification of beating accessed healthcare services than women who justified one or more reasons. This gradient is relatively linear for participants who justified three or less reasons and slightly convoluted for those who justified four or more reasons. Across three categories of attitude on beating (i.e. reject: strongly, moderately and weakly) there were downward trends in terms of the number of women who responded ‘affirmative’ on each of the seven items of healthcare utilization (results not shown).

The relationship between selected demographic variables and healthcare seeking behaviors are presented in Table 2. Educational status of women and their husbands, and economic status of the household had a significantly positive association with the utilization of healthcare services by the participants. Urban women were more likely than rural women to report using all except one of the healthcare services.

**Table 2:** Adjusted odds ratio of the association between major demographic variables and study outcomes.

	Used contraceptive (95% CI)	Used modern contraceptive (95% CI)	Made ≥1 ANC visit (95% CI)	Made ≥4 ANC visit (95% CI)	Delivered in healthcare facilities (95% CI)	Reported delivery by SHP (95% CI)	Reported postnatal checkup by SHP (95% CI)
Age	1.03 (1.02–1.03)	0.93 (0.92–0.94)	0.97 (0.96–0.97)	0.97 (0.96–0.98)	0.99 (0.98–1.00)	0.97 (0.96–0.98)	0.97 (0.96–0.98)
Urban (ref rural)	1.61 (1.46–1.76)	0.92 (0.75–1.13)	1.24 (1.34–1.37)	1.58 (1.42–1.77)	1.78 (1.61–1.97)	1.57 (1.43–1.72)	1.53 (1.39–1.69)
Participant’s year of education	1.03 (1.02–1.05)	1.03 (1.00–1.06)	1.09 (1.08–1.11)	1.11 (1.09–1.13)	1.14 (1.13–1.16)	1.12 (1.11–1.14)	1.11 (1.09–1.13)
Husband’s year of education	1.00 (0.99–1.01)	0.98 (0.96–1.00)	1.04 (1.03–1.05)	1.03 (1.02–1.05)	1.04 (1.03–1.06)	1.04 (1.03–1.05)	1.04 (1.03–1.05)
Economic status							
-Richest	0.99 (0.86–1.13)	1.41 (1.03–1.93)	3.12 (2.70–3.62)	3.30 (2.66–4.09)	3.91 (3.29–4.63)	3.42 (2.93–4.00)	3.65 (3.07–4.34)
-Richer	0.94 (0.84–1.05)	1.40 (1.08–1.82)	2.23 (1.97–2.52)	2.21 (1.80–2.70)	2.31 (1.98–2.69)	2.36 (2.05–2.71)	2.29 (1.95–2.69)
-Middle	1.07 (0.97–1.19)	1.06 (0.83–1.33)	1.60 (1.42–1.79)	1.53 (1.25–1.89)	1.61 (1.38–1.87)	1.66 (1.44–1.91)	1.64 (1.40–1.93)
-Poorer	1.23 (1.13–1.36)	1.10 (0.88–1.38)	1.30 (1.16–1.46)	1.34 (1.08–1.13)	1.32 (1.13–1.54)	1.35 (1.17–1.56)	1.26 (1.07–1.49)
-Poorest (ref)	1.00	1.00	1.00	1.00	1.00	1.00	1.00

Note. ANC: antenatal care, SHP: skilled health professionals

Table 3 shows unadjusted and adjusted odds ratios (ORs) and their corresponding confidence intervals (CIs) for respondents’ healthcare seeking behavior. Participants’ attitudes towards wife-beating have a significant association with each of these six forms of healthcare seeking behavior except for modern contraception use. Women who reported moderate and strong rejection towards wife-beating were 1.21 times (95% CI, 1.01–1.44) and 1.19 times (95% CI, 1.02–1.39) more likely to report any contraception use, respectively. Additionally, women who strongly rejected the justification of wife beating were more likely to report accessing healthcare than those who either rejected moderately or weakly. For instance, women who rejected this abuse moderately and strongly were 1.21 times (95% CI, 1.00–1.48) and 1.35 times (95% CI, 1.13–1.63) more likely to report making ANC visits than women who rejected weakly, respectively. Compared to the women who rejected weakly, the odds of using a health facility during delivery was 1.55 times (95% CI, 1.22–1.98) high for women who rejected strongly. This relationship was elevated (aOR, 1.21; 95% CI, 0.93–1.58) for the respondents who moderately rejected the justification for wife-beating. Compared to the weakly rejecting group, strong rejecters were 1.55 times (95% CI, 1.20–2.01) and 1.52 times (95% CI, 1.22–1.89) likely of getting postnatal checkups and delivery by SHP. These results were elevated for the respondents who moderately rejected the reasons for wife-beating.

**Table 3:** Association between healthcare-seeking behaviors and maternal attitudes towards wife-beating in Bangladesh, 2011–2014 (n = 9,632).

Outcome variable	Attitude toward wife-beating		
	Reject Weakly	Reject Moderately	Reject Strongly
**Used contraceptive**			
n (%)	259 (59.7)	816 (62.0)	4909 (63.9)
OR (95% CI)	1.00	1.26 (1.06–1.49)	1.30 (1.12–1.52)
aOR (95% CI)	1.00	1.21 (1.01–1.44)	1.19 (1.02–1.39)
**Used modern contraceptive**			
n (%)	233 (91.0)	758 (92.9)	4586 (93.4)
OR (95% CI)	1.00	1.00 (0.67–1.48)	1.12 (0.78–1.61)
aOR (95% CI)	1.00	1.00 (0.67–1.51)	1.01 (0.70–1.46)
**Made ≥1 ANC visit**			
n (%)	171 (41.0)	649 (51.1)	4708 (63.6)
OR (95% CI)	1.00	1.44 (1.19–1.74)	2.07 (1.74–2.46)
aOR (95% CI)	1.00	1.21 (1.00–1.48)	1.35 (1.13–1.63)
**Made ≥4 ANC visit**			
n (%)	38 (16.3)	217 (25.9)	2015 (35.6)
OR (95% CI)	1.00	1.69 (1.19–2.41)	2.75 (1.98–3.80)
aOR (95% CI)	1.00	1.35 (0.94–1.95)	1.59 (1.14–2.23)
**Delivered in healthcare facilities**			
n (%)	82 (18.6)	1008 (75.1)	2964 (37.8)
OR (95% CI)	1.00	1.49 (1.16–1.91)	2.75 (2.19–3.46)
aOR (95% CI)	1.00	1.21 (0.93–1.58)	1.55 (1.22–1.98)
**Reported delivery by SHP**			
n (%)	94 (21.3)	382 (28.4)	3287 (41.9)
OR (95% CI)	1.00	1.48 (1.18–1.87)	2.57 (2.08–3.17)
aOR (95% CI)	1.00	1.20 (0.94–1.53)	1.52 (1.22–1.89)
**Reported postnatal checkup by SHP**			
n (%)	73 (17.5)	289 (22.8)	2703 (36.5)
OR (95% CI)	1.00	1.47 (1.12–1.92)	2.65 (2.08–3.39)
aOR (95% CI)	1.00	1.16 (0.88–1.55)	1.55 (1.20–2.01)

Note. aOR: adjusted odds ratio, ANC: antenatal care, SHP: skilled health professionals, CI: confidence interval

## Discussion

Our findings suggest respondents’ attitudes towards wife-beating were a significant factor for reproductive healthcare seeking behavior. If we consider women’s attitudes towards wife-beating a proxy to the real-life abuse, this finding has substantial public health implications. Noticeably, this abuse (or the proxy) is a barrier to accessing several forms of essential healthcare that are likely to impact on health and well-being of participants and their children. Elimination and/or prevention of gender norms that cause this ill-practice should be the primary focus of policy intervention [26, 27]. However, this is a delicate issue, which demands careful intervention. Therefore, the gradual development of social momentum in opposition to spousal abuse is crucial.

Over the recent decades violence against women has shifted significantly from being considered a private or family problem to being recognized as a social and public health concern with serious consequences for health and wellbeing of the victims [28, 29]. Adverse health impact of abused women was reported by a number of previous studies from various international settings [30–33]. The findings from this study now adds evidence to the existing body of literature that women who justify reasons for spousal abuse may also suffer from similar adverse health outcomes, as they access necessary reproductive care much less than others.

### Geographical variation

We found geographical variation in our results–both in terms of the extent to which abuse is justified and healthcare utilization. A number of previous studies also consistently reported spatial variation in the prevalence of domestic violence [34] and the acceptance of IPV within the marriage [35]. We also found variation between urban and rural areas. This spatial variation is likely due to the variation across regions in terms of social norms and practices, which are deeply rooted and strongly influenced by and intermingled with a range of factors such as education, economic status, employment, culture, religion, to name a few [36, 37]. When considered from a policy perspective, this variation suggests a necessity to implement geographically tailored interventions.

### Possible reasons for justifying wife-beating

This study does not examine the underlying causes of justifying wife-beating and their association with reproductive healthcare seeking behavior. Further studies are needed for identifying the causal pathways. Some information on this issue, however, is available in the existing literature. For instance, Vung et al (2008) [38] identified a range of factors associated with IPV and categorized them into four main groups: individual, relationship, community and societal. This, and a few other studies, report that IPV is likely to pass through generations [38–40]. Women who witness partner violence during their childhood are more likely to report experiencing IPV in their own adult life, and they also seem to hold more tolerant attitudes towards violence [38]. The literature suggests that violence is frequently used to resolve a crisis of male identity. Risk of violence is greatest in societies where the use of violence is socially accepted [41]. Women who are more empowered educationally, economically, and socially are most protected, but below this high level the relation between empowerment and risk of violence is known to be non-linear [41].

### Likely causal pathways

A relatively low rate of healthcare utilization among women who justified several reasons for wife-beating could be explained by the fact that this subgroup lacks empowerment and the sense of entitlement. A lower score on the “number of reasons wife-beating is justified” indicates a greater sense of entitlement, self-esteem and status that reflect positively on their sense of empowerment and ability to claim their rights [11, 42]. Perhaps a good example to support this assumption is our finding about the higher prevalence of contraception use among the women who moderately or strongly reject the reasons for wife-beating. This is likely to be an indication of women’s ability to negotiate safer sexual practices. This ability is crucial for making a decision of seeking and or utilizing healthcare.

There is a negative trend in utilization of healthcare services across three categories of attitude on abuse (i.e. reject strongly, reject moderately and reject weakly). This trend has important public health implications. An improvement in women’s awareness about their rights and their attitudes against abuse are likely to increase their healthcare seeking behaviors. Most of our public health interventions often pay considerable importance towards making services available. Although availability is an important aspect, some previous studies suggest available services may not be accessed by women who lack self-esteem or are subject to societal dominance of gender norms [43–45]. Thus, eradication of this norm and primary prevention of violence are essential. However, in reality, any effort to this end is often over-shadowed by the importance of the large number of programs that, understandably, seek to deal with the immediate and numerous consequences of violence [46].

In the adjusted models, some socio-economic factors such as educational levels of women and their husbands, economic status of the household and geographical locations were found to be significantly associated with the level of healthcare utilization. This observation is mostly consistent with the findings of previous studies in Bangladesh [47–49] and other developing countries [50, 51]. Formal education, economic status and living environment–all are intertwined and an improvement in any of these factors are likely to empower women and their access to basic healthcare services. Although this improvement needs multifaceted endeavors from all fronts, from the policy perspective perhaps a special focus on ensuring longer years of formal education for women is the most important and achievable way forward [52, 53].

### Strengths and limitations

Our study has several strengths and some limitations. We used the two most recent nationally representative datasets, which yielded a large sample collected from the entire country. Furthermore, we adjusted our models for a wide range of confounders and this enhanced the reliability of our findings. However, women’s attitudes towards wife-beating was considered a proxy to direct violence. It is not unlikely that some women hold a poor attitude towards abuse although they are not abused by their husbands. Moreover, the questions only assessed attitudes towards one specific form of physical abuse ‘wife-beating' and did not enquire about other forms of abuse (e.g. sexual and or emotional abuse), which may result in an underestimation of attitudes towards accepting violence against women. This study examined cross-sectional data, therefore, the relationship is correlational only. Also, the data were based on participants’ self-reporting with no scope of validation by interviewers that may be subject to reporting error. Lastly, despite taking precautionary measures to protect privacy, it is still possible that some women did not disclose their true attitudes towards wife-beating. Moreover, utilization of healthcare is subject to the availability of and accessibility to services. The distance between healthcare services and women’s residence might be another important factor, which we were unable to adjust due to lack of information.

## Conclusions

One-third of the women justified hitting or beating by their husband in particular circumstances. This attitude towards violence was found to be a significant factor for the utilization of basic healthcare services. Women who strongly rejected the justification of wife-beating were more likely to report the utilization of basic healthcare services than women who rejected that moderately or poorly. Although this observed relationship is co-relational only, this has important policy implications. Social changes are essential to improve women’s attitudes against intimate partner violence, and to eradicate the existing gender and cultural norms that motivate the malpractice of wife-beating. The impact of this abuse on women’s health needs to be brought to the forefront of public health and health promotion campaigns. Findings of this study should also inform mainstream health services, specialist women’s services and support agencies.

## Abbreviations

IPV: Intimate partner violence
PTSD: Posttraumatic stress disorder
BDHS: Bangladesh Demographic and Health Survey
SHPs: Skilled health professionals
ANC: Antenatal care
OR: Odds Ratio
aOR: Adjusted odds ratio
WHO: World Health Organization
